# EASIX Is an Accurate and Easily Available Prognostic Score in Critically Ill Patients with Advanced Liver Disease

**DOI:** 10.3390/jcm12072553

**Published:** 2023-03-28

**Authors:** David Schult, Sebastian Rasch, Roland M. Schmid, Tobias Lahmer, Ulrich Mayr

**Affiliations:** Medizinische Klinik und Poliklinik II, Klinikum rechts der Isar, Technische Universität München, Ismaninger Straße 22, D-81675 München, Germany

**Keywords:** EASIX, liver cirrhosis, acute-on-chronic liver failure, endothelial dysfunction, intensive care unit

## Abstract

Acute-on-chronic liver failure (ACLF) is associated with high mortality. Objective prognostic scores are important for treatment decisions. EASIX (Endothelial Activation and Stress Index) is a simple biomarker consisting of LDH, platelets, and creatinine, reflecting endothelial dysfunction after allogeneic stem cell transplantation. Considering endothelial dysfunction in the pathogenesis of ACLF, this study aimed to test the discriminative ability of EASIX in advanced liver disease. We retrospectively analysed the prognostic potential of EASIX to predict 28-day and 3-month mortality in a total of 188 liver cirrhotic patients requiring treatment at the intensive care unit. We evaluated the ability of EASIX to rule out early infections and predict the need for hemodialysis. EASIX performed moderately better than established scores in predicting 28-day mortality (AUC = 0.771) and was nearly equivalent (AUC = 0.791) to SOFA and APACHE-II in the prediction of 3-month mortality. Importantly, EASIX showed better diagnostic potential in ruling out clinically apparent infections than common proinflammatory markers (AUC = 0.861, *p* < 0.001) and showed suitable accuracy in predicting the need for hemodialysis (AUC = 0.833). EASIX is an accurate, objective and easily assessable biomarker for predicting mortality and complications in patients with advanced liver disease.

## 1. Introduction

Liver cirrhosis (LC) is an increasing cause of mortality and morbidity in affluent countries and advanced liver disease is a challenging disorder especially in critically ill patients [1]. Impairment of hepatocellular function, hepatic encephalopathy (HE), portal hypertension and ascites are hallmarks of decompensation [2,3,4,5]. Decompensated liver cirrhosis is frequently complicated by acute-on-chronic liver failure (ACLF), which is characterised by intense systemic inflammation and sequential multi-organ failure with high mortality [3,4,5] requiring therapy at an intensive care unit (ICU). Although the pathophysiology of LC is multifactorial, there are common etiological characteristics including inflammation, angiogenesis, stimulation of hepatic stellate cells with consecutive fibrogenesis, hepatic sinusoidal remodelling and hepatic endothelial dysfunction (ED) [6,7]. The circumstances leading to decompensation of LC and ACLF are not conclusively understood. However, there is growing evidence that systemic inflammation [8] and ED of sinusoidal endothelial cells (SEC) promote fibrosis and ACLF [9,10,11,12,13,14].

In this context, ED is characterised by an impaired balance between vasoconstrictors and vasodilators. This results in an increased hepatic vascular tone proceeding the development of portal hypertension and, on the other hand, an increased splanchnic blood flow leading to hyperdynamic circulatory syndrome [11,15]. Portal hypertension, in turn, has multiple clinical consequences, including an increased risk of varicose hemorrhage and ascites. Hyperdynamic circulatory syndrome results in persistent arterial hypotension. Furthermore, SEC in the cirrhotic liver show impaired filtration barrier with consecutively altered hepatic metabolism [16] which may contribute to HE and ACLF.

To predict complications and mortality in patients with LC, reliable and reproducible clinical scores are of vital importance for individualised treatment. Scores for advanced liver cirrhosis and ACLF in the intensive care setting often contain parameters that are assessed more subjectively. These include, for example, the Glasgow coma scale or classification of hepatic encephalopathy. EASIX (Endothelial Activation and Stress Index) is a simple scoring system obtained by the following formula: (lactate dehydrogenase [LDH, U/L] × creatinine [mg/dL])/thrombocytes [10^9^ cells per L]. The score has been shown to be associated with biomarkers of endothelial homeostasis and inflammatory response such as angiopoietin-2 [17], interleukin-18, CXCL8, and insulin-like growth factor 1 [18] and was established to predict mortality and endothelial complications after allogeneic stem cell transplantation (alloTPL) [18] and acute graft-versus-host disease (GvHD) [19]. Furthermore, the score predicts the onset of hepatic sinusoidal obstruction syndrome [20] and is correlated with early hyperbilirubinemia after alloTPL [17]. Because EASIX considers ED, which seems to be crucial in the pathophysiology of LC and decompensation [9,10,11,12,13,14], we propose EASIX as an easily to perform and objective biomarker for mortality and highly relevant complications in patients with end-stage liver disease.

In this study, we primarily analysed the prognostic accuracy of EASIX to predict mortality in patients with LC and ACLF admitted to the ICU. The predictive value of EASIX was compared to established scores, specifically MELD and CTP as well as APACHE-II (acute physiology and chronic health evaluation) and SOFA (sequential organ failure assessment). As secondary endpoints, we elucidated the diagnostic ability of EASIX to exclude apparent infections upon transfer to ICU and to predict the indication for hemodialysis therapy.

## 2. Material and Methods

### 2.1. Study Design

We retrospectively screened all patients with liver cirrhosis admitted to our ICU between January 2016 and May 2022. Treatment and diagnostic procedures followed current standards in our ICU, irrespective of this study. The diagnosis of liver cirrhosis was based on the following criteria: medical reports suggesting end-stage liver disease (i.e., variceal bleeding, episodes of ascites, or HE), imaging methods with typical morphological criteria and/or histopathological characteristics of cirrhosis and laboratory disorders indicative of an impaired liver function in presence of risk factors for cirrhosis.

Patients with incomplete knowledge about clinical or laboratory findings to assess the scores evaluated in this study were excluded (N = 3). In addition, patients who underwent liver transplantation within the observation period (N = 3) and cirrhosis with hepatocellular carcinoma (N = 3) were excluded because of the obvious influence on outcome. Furthermore, patients lost during the observation period (N = 2) and patients re-admitted after prior ICU treatment in our hospital (N = 3) were not included in the study.

We enrolled 192 patients with LC admitted to our ICU for assessment of baseline EASIX score. Prior dialysis therapy due to terminal renal insufficiency was pre-existing in 4 patients and since creatinine is a key variable in calculation of EASIX, these patients were excluded. Finally, prognostic analyses of EASIX were performed in a total of 188 patients. An overlapping proportion of 34% of the current population (N = 64) was included in a previous study from our centre [21]. Certain methods used in the assessment of laboratory and infectious parameters as well as the statistical analysis were previously published by our group [21].

### 2.2. Evaluation of Clinical Scores

The calculation of clinical scores were performed as described in [21]. Blood samples were collected irrespective of this study following the current standards in our ICU. We calculated EASIX retrospectively from laboratory results on the day of admission using the formula (lactate dehydrogenase [LDH, U/L] × creatinine [mg/dL]/thrombocytes [10^9^ cells per L]) as described previously [19]. Laboratory results were used for calculation of scores defining the health status of patients, such as the SOFA- and APACHE-II scores. Parameters of liver function were used for the evaluation of cirrhosis in the context of CTP and MELD scores. Definition of ACLF was based on recommendations from the EASL CLIF consortium [22], discriminating between no ACLF and ACLF-Grade I–III. Clinical parameters used to asses organ function, namely international normalised ratio (INR), creatinine, white blood cell count (WBC), HE, mean arterial pressure (MAP), use of vasopressors, arterial partial pressure of oxygen (P_a_O_2_), fraction of inspired oxygen (F_i_O_2_) or need for mechanical ventilation were considered for the calculation of CLIF acute-on-chronic liver failure score (CLIF-ACLF) and CLIF organ failure score (CLIF-OF). Thereby, HE was defined according to the West Haven criteria [23]. Moreover, we assessed the common inflammatory parameters C-reactive protein (CRP) as well as procalcitonin (PCT). The evaluation of CRP, PCT and WBC is an institutional routine practice to identify proinflammatory reactions or infectious complications. Time of laboratory analysis was 60–120 min. Laboratory tests were performed by the department of clinical chemistry of our hospital.

### 2.3. Analyses Regarding Presence of Infection

On admission to ICU, all patients were routinely screened for the presence of infection. Two pairs of blood cultures were collected to exclude bloodstream infections. Urine sediment and cultures were examined for the presence of urinary tract infections. Chest X-ray or computed tomography (CT) were used to exclude or diagnose respiratory infections. Broncho-alveolar lavage (BAL) was performed in patients with mechanical ventilation to identify specific pathogens of bronchoalveolar infections. To find bacterial pathogens of SBP in case of decompensation, specimens of ascites were inoculated into anaerobic and aerobic blood culture bottles after paracentesis.

These tests allowed further classification of our cohort. Bacteremia was confirmed by the presence of at least one positive blood culture. Urinary tract infection was diagnosed in case of either positive urine culture or pathological dipstick/sediment. Patients with either radiological signs of pneumonia or evidence of pathogens in BAL were diagnosed as having pulmonary infection. Ascitic polymorphonuclear neutrophils (PMN) ≥ 250/µL or ascitic cultural isolation of bacteria was diagnostic for spontaneous bacterial peritonitis (SBP).

Patients meeting at least one pathological finding regarding the presence of infection as described were classified as having *evidence of infection (“Infection”)*. On the contrary, patients with *no evidence of infection* on admission to ICU were categorised as “*No Infection*” as previously described in [21].

### 2.4. Collection of Data

All clinical, laboratory and individual parameters necessary for the calculation of SOFA, APACHE II, MELD, CTP, ACLF, CLIF-OF, CLIF-ACLF and EASIX scores were obtained from the day of admission to ICU. Patients were observed until death or a minimum of 3 months for survival analyses 28 days and 3 months after admission to the ICU.

### 2.5. Statistical Analysis

Statistical analyses were conducted as previously published by our group [21]. Continuous variables are shown as median and interquartile range (IQR). Categorical variables are presented as percentages. A nonparametric, two-tailed Mann–Whitney test was used to compare patient cohorts. Correlation analyses were performed using Spearman’s coefficient r_s_ and linear regressions using the coefficient R^2^. Receiver operating characteristic curves (ROC) were used to represent the prognostic potential of EASIX and other parameters with respect to 28-day and 3-month mortality via the area under the curve (AUC). Appropriate cut-offs were determined using Youden’s index, considering the highest combined sensitivity and specificity. Moreover, positive predictive value (PPV) and negative predictive value (NPV) were calculated to further specify the diagnostic and prognostic ability of EASIX. Survival analyses were performed according to the Kaplan–Meier method and all deaths were considered as events. Log rank (Mantel–Cox) test was used for comparison of survival curves. Associations of individual variables with the risk of mortality were reported as hazard ratio (HR) according to Mantel–Haenszel. A *p*-value < 0.05 was considered significant. Analyses and graphs were generated by using GraphPad Prism 8.0 (GraphPad Software, La Jolla, CA, USA).

## 3. Results

### 3.1. Characteristics of the Study Population

Overall, 188 patients were included, consisting of 69 female and 119 male patients. The baseline characteristics of the study population and clinical scores are shown in Table 1.

### 3.2. Prognostic Ability of EASIX

**Figure 1:**
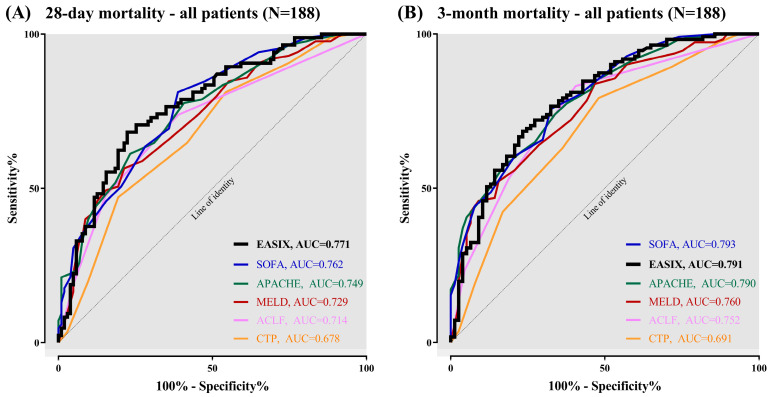
Prognostic accuracy of EASIX versus clinical scores to predict mortality in patients with advanced liver disease (N = 188): (**A**) 28-day mortality; and (**B**) 3-month mortality.

We used ROC curves to predict outcomes of patients with liver cirrhosis transferred to the ICU: In the total study population of 188 patients, baseline EASIX revealed a suitable prognostic accuracy to predict 28-day mortality (AUC = 0.771). As depicted in Figure 1A, EASIX performed moderately better than SOFA (AUC = 0.762), APACHE-II (AUC = 0.749), MELD (AUC = 0.729), and ACLF Grade I–III (AUC = 0.714) with the greatest improvement to CTP (AUC = 0.678). We found a PPV of 72.7% and a NPV of 63.2% in the prediction of 28-day mortality.

Analogously, Figure 1B displays the ROC curves for the prediction of outcome 3 months after admission to the ICU. The prognostic accuracy of EASIX (AUC = 0.791) was nearly equivalent to SOFA (AUC = 0.793) and APACHE-II (AUC = 0.790), whereas prognostic potential was superior to MELD (AUC = 0.760), ACLF Grade I–III (AUC = 0.752) and CTP (AUC = 0.691). Further evaluations revealed a PPV of 77.1% and a NPV of 65.1% to predict 3-month mortality.

**Figure 2:**
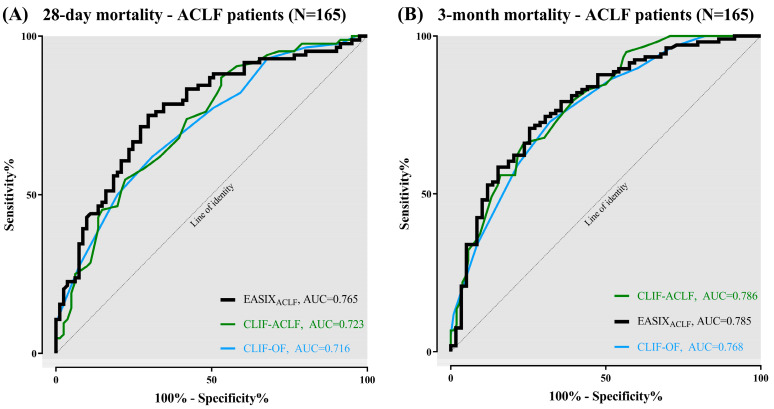
Prognostic ability of EASIX in ACLF-patients (N = 165) versus CLIF-OF and CLIF-ACLF to predict mortality: (**A**) 28-day mortality; and (**B**) 3-month mortality.

Moreover, we analysed the prognostic power of baseline EASIX in the subgroup of 165 patients fulfilling criteria of ACLF on ICU admission. Concerning 28-day mortality (Figure 2A), EASIX (AUC = 0.765) had a prognostic advantage compared with CLIF-ACLF (AUC = 0.723) and CLIF-OF (AUC = 0.716) with a PPV of 77.8% and a NPV of 61.7%. Regarding 3-month mortality (Figure 2B), the prognostic ability of EASIX (AUC = 0.785) was equivalent to CLIF-ACLF (AUC = 0.786) and superior to CLIF-OF (AUC = 0.768). Analyses resulted in a PPV of 68% and NPV 83.3% for baseline EASIX to predict 3-month mortality.

### 3.3. Survival Analyses Based on Baseline EASIX

**Figure 3:**
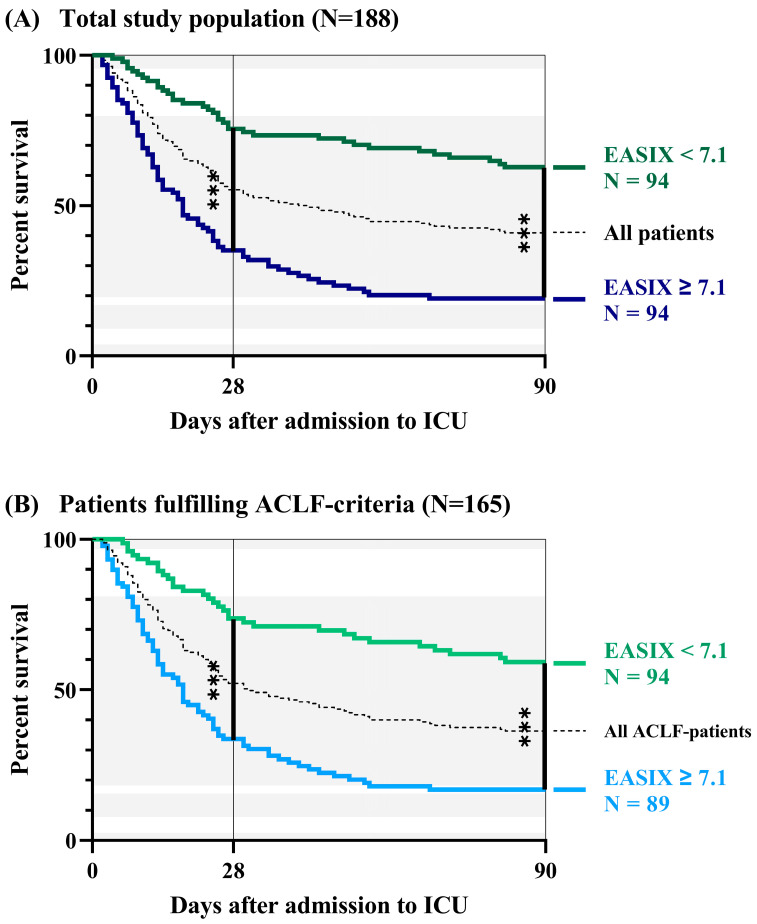
Survival analyses based on EASIX on admission to the ICU. (**A**) Total study population of 188 patients (baseline levels of EASIX ≤ 7.1 versus > 7.1); and (**B**) subgroup of 165 patients fulfilling ACLF criteria (admission levels of EASIX ≤ 7.1 versus > 7.1). *** = *p* < 0.001.

Based on ROC analyses, we performed survival analyses depending on levels of EASIX on admission to our ICU. In the total study cohort of 188 patients, EASIX revealed a sensitivity of 68.5% and a specificity of 76.6% to predict 3-month mortality with a cut-off for EASIX ≥ 7.1. As depicted in Figure 3A, baseline EASIX ≥ 7.1 was associated with a significantly increased risk of 3-month mortality compared with patients with baseline EASIX < 7.1 (HR 3.96, 95% CI = 2.67–5.87, *p* < 0.001).

Subgroup analysis in 165 patients fulfilling criteria of ACLF on ICU admission resulted in a sensitivity of 70.8% and a specificity of 74.6% for the prediction of 3-month mortality with a cut-off for EASIX ≥ 7.1. Analogously, admission levels of EASIX ≥ 7.1 in ACLF patients were linked to a significantly increased risk of mortality compared with ACLF patients with baseline EASIX < 7.1 (HR 3.63, 95% CI = 2.44–5.43, *p* < 0.001; Figure 3B).

A significant proportion of about 88% was classified as fulfilling ACLF criteria on admission to the ICU. Consequently, survival curves of the total study population (N = 188) and ACLF patients (N = 165) were nearly congruent. However, the overall 3-month survival rate was even lower in the ACLF-group (36%) compared with the total cohort (41%). As expected, the presence of ACLF on admission to ICU was related to a significantly increased risk for 3-month mortality compared with patients without ACLF (HR 2.40, 95% CI = 1.44–4.01, *p* = 0.002).

### 3.4. Diagnostic Potential of EASIX

**Figure 4:**
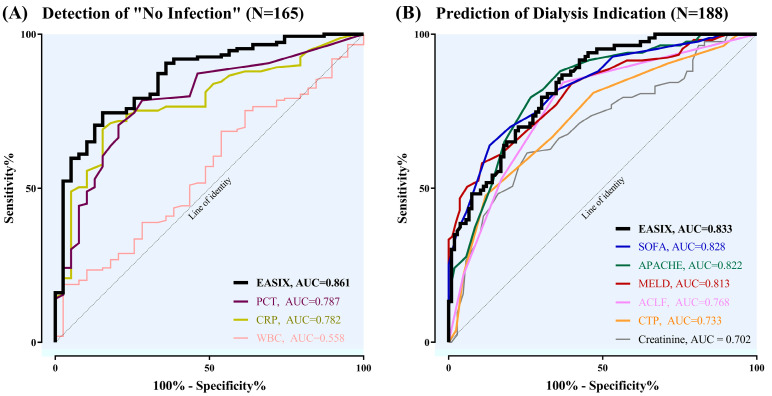
Diagnostic ability of baseline EASIX. (**A**) Diagnostic ability of baseline EASIX to rule out clinically overt infections (“*No Infection*”) on ICU admission in comparison with PCT, CRP and WBC. (**B**) Predictive value of baseline EASIX compared with common clinical scores in identifying patients requiring hemodialysis therapy during their ICU stay.

Further ROC analyses were performed to elucidate the diagnostic ability of baseline EASIX to rule out clinically apparent infections in critically ill patients with cirrhosis. A large proportion of approximately 79% of the total study population was classified as having “*Infection*” (N = 149), while only 21% had no evidence of infection on admission to ICU (N = 39). The “*Infection*” group revealed much higher EASIX than “*No Infection*” (*p* < 0.001). The distribution of various types of infectious diseases with corresponding levels of EASIX on admission is demonstrated in Supplementary File S1. EASIX showed a high diagnostic sensitivity of 84.6% and specificity of 74.5% with cut-off levels ≤ 4.9 to identify patients categorised as “*No Infection*” at the day of admission to the ICU. As demonstrated in Figure 4A, EASIX (AUC = 0.861) was superior to the conventional inflammatory markers PCT (AUC = 0.787), CRP (AUC = 0.782) and WBC (AUC = 0.558). We found a NPV of 87.5% and a PPV of 82.2% for EASIX to rule out infectious diseases on ICU admission. Concerning the discriminative ability of the single components of EASIX, baseline values of creatinine (AUC = 0.753) and thrombocytes (AUC = 0.734) performed better in identifying patients with “*No Infection*” compared with LDH (AUC = 0.695).

Additionally, we evaluated the potential of baseline EASIX to diagnose the necessity for hemodialysis therapy within 90 days from admission to the ICU in comparison with common clinical scores. According to ROC curves, EASIX revealed a suitable accuracy in identifying critically ill cirrhotic patients requiring hemodialysis during their ICU stay (AUC = 0.833), with a PPV of 81.6% and a NPV of 66.3%. We found a corresponding sensitivity of 63.2% and a specificity of 86.8% with a cut-off ≥ 9.5. As illustrated in Figure 4, EASIX was nearly equivalent to SOFA (AUC = 0.828), APACHE-II (AUC = 0.822) and MELD (AUC = 0.813), whereas its accuracy was superior to ACLF grade I– III (AUC = 0.768), CTP (AUC = 0.733) and baseline creatinine (AUC = 0.702). Furthermore, we used ROC curves to analyse the accuracy of the single parameters included in the EASIX calculation for the prediction of haemodialysis; we found a higher diagnostic ability of thrombocytes (AUC = 0.739) and creatinine (AUC = 0.700) in comparison with LDH (AUC = 0.665).

### 3.5. Correlation Analyses

Correlations and linear regressions of baseline EASIX with various clinical scores and proinflammatory parameters are listed in Table 2. EASIX was not correlated with creatinine, as the latter is an essential part of EASIX calculation.

## 4. Discussion

This study primarily demonstrated a suitable prognostic accuracy of EASIX in critically ill patients with LC and ACLF.

The parameters used in the EASIX formula are routine markers of thrombotic microangiopathy as a form of ED in the setting of alloTPL [19]. Considering the importance of ED in GvHD and cardiovascular complications in hematologic malignancies, EASIX was consecutively established as a prognostic score in patients after alloTPL [19] and patients with myelodysplastic syndromes [24].

Alterations in the function of endothelial cells are considered to be of importance in many critical illnesses [25]. For example, a key role of ED in the development of acute respiratory distress syndrome in coronavirus disease 2019 (COVID-19) is hypothesised, and EASIX was shown to be a predictive marker of outcomes in COVID-19 [26]. In addition, EASIX is attractive because of its simplicity and the laboratory parameters of the score are routinely used in the diagnosis of various clinical conditions, especially malignant diseases. Indeed, the score has been shown to be an independent prognostic factor for the survival of patients with diffuse large B-cell lymphomas [27]. Furthermore, EASIX is associated with biomarkers of inflammatory response in critically ill patients who have undergone alloTPL [17,18].

Inflammation and oxidative stress are hallmarks of decompensation and ACLF [2], leading to a reduced nitric oxide (NO) production and further ED in SEC [11].

In addition to the importance of ED and systemic inflammation in the pathogenesis of ACLF, the laboratory parameters used in the EASIX score have independent relevance in the diagnosis of advanced liver disease. Patients with chronic liver disease usually show reduced platelet counts due to splenic sequestration and bone marrow insufficiency [28]. In this context, thrombocytopenia often correlates with the prognosis in patients with liver cirrhosis [29,30]. Creatinine serves as the most important parameter of acute renal failure and hepatorenal syndrome, which are frequent complications of liver cirrhosis and ACLF [31]. LDH is less commonly used in routine diagnosis of advanced liver disease. However, LDH is a marker of cell damage and prognostic in various critical illnesses [32,33]. Furthermore, it has been shown that acute liver injury increases the hepatocytic activity of LDH in mice, which may have potential therapeutic consequences [34].

Considering the relevance of excessive inflammatory response and ED in the pathogenesis of ACLF, EASIX may be a potentially useful marker to assess prognosis and complications in end-stage liver disease.

According to the present results, EASIX was superior to established clinical scores in predicting short-term mortality (Figure 1A and Figure 2A) and showed sufficient results in predicting 3-month mortality (Figure 1B and Figure 2B). Compared with CTP and MELD score, EASIX showed better results in predicting mortality in all patients. Here, an EASIX score of ≥7.1 was associated with significantly increased mortality in both the cohort of all patients (N = 188) and the ACLF subgroup (N = 165) (Figure 3A). The cut-off of ≥7.1 in our cohort is comparable to the median EASIX in patients after chemotherapy [17]. The relatively high score in our cohort of critically ill patients is mainly due to the relatively low platelets combined with high creatinine (Table 1), typically observed in patients with end-stage liver cirrhosis and ACLF. Values for LDH were on average within the normal range and LDH contributed least overall to the EASIX score. However, it can be assumed that with a longer intensive stay and greater disease severity, LDH increases and contributes relevantly to the EASIX score when collected dynamically. This is because LDH has been proposed as a marker of cell death and hypoxia in various clinical conditions, including sepsis and HE [35,36]. Furthermore, LDH activity was found to be elevated in hepatocytes of mice with acute liver failure, contributing to apoptosis [34]. Besides its association with ED within the EASIX formula, LDH is considered to reflect this condition of liver damage in ACLF. Thrombocytes and creatinine are particularly critical components of EASIX in the specific population of ICU patients with advanced liver disease. However, the involvement of LDH seems to contribute to the prognostic and diagnostic ability of EASIX. The extent to which the different parameters of EASIX are associated with ED in general was described in the original publication [18]. The pathophysiologic causes of ED in end-stage liver disease and hematologic neoplasms may differ. Nevertheless, the underlying mechanisms of ED in critically ill patients are comparable.

Besides accurately predicting mortality, baseline EASIX is also able to identify LC patients with evidence of bacterial infections at the day of admission to the ICU. Furthermore, EASIX showed a high discriminative ability to predict the need for hemodialysis within the ICU stay (Figure 4).

Bacterial infections are considered a major cause and complication of ACLF [5,37], although the infection may not always be detected in the first place, leading to a delay in antibiotic therapy. High levels of inflammation are typical for ACLF [2]. CRP and PCT are the most commonly used proinflammatory parameters to detect infectious complications [38,39]. However, in patients with severe cirrhosis, the increase in CRP is often reduced, pointing to a defective acute phase reaction in advanced liver disease and limiting the prognostic value of CRP in the intensive care setting [40,41]. This highlights the importance of independent biomarkers that can indicate infection at a very early stage. A clinical parameter that can identify patients at risk for consecutive infections at an early stage of ACLF may provide a rationale for prompt antibiotic therapy and potentially improve survival. Interestingly, EASIX performed better than conventional inflammatory markers in ruling out clinically overt infectious diseases (Figure 4A). Further studies are needed to evaluate whether antibiotic therapy on the background of an elevated EASIX can reduce the rate of infectious complications in patients with ACLF.

Besides a decreased renal perfusion, inflammation of the kidney with consecutive impaired renal microcirculation is thought to trigger acute kidney injury (AKI) in LC with systemic inflammatory response [31]. AKI was found to be within the most frequent organ failures in ACLF, contributing significantly to mortality [4]. The timing of dialysis for bridging to liver transplantation is not fully known [31]. EASIX is easy to calculate and suitable for early screening of patients who potentially need renal replacement therapy. Early scores to identify patients at risk for complications can be important for individualised treatment strategies and counselling of patients and families. Thereby, early parameters enable physicians to better plan the next steps in the context of time-critical diseases such as ACLF. Patients and relatives can be informed more objectively about the prognosis based on scores and can be involved in decision-making processes. However, it is important to note that clinical decisions and counselling of patients should not be based on scores alone and always be considered in a comprehensive perspective.

Established scores for prognostic assessment of ACLF are often time consuming to collect and contain subjective parameters. The SOFA score, for example, considers the Glasgow coma scale, which is often collected at first in the ICU setting, when patients are intubated, rather than at initial presentation to the emergency department. The CLIF-ACLF score contains a variety of parameters, including the assessment of HE, which partly follows subjective criteria and is not easy to classify, especially for inexperienced physicians. MELD includes INR and therefore is affected by variability and interlaboratory variation of INR determinations. EASIX consists of only three routine laboratory parameters (LDH, creatinine, thrombocytes), making it easy to collect, cost effective, and objective.

Although the results are conclusive and statistically significant, our study has important limitations. It is a single-centre study performed exclusively in the ICU setting and thus evaluated a limited number of patients. Furthermore, patients with LC were not compared to other critically ill patients. The present study did not assess dynamic scores acquired during the course of intensive stay and focused exclusively on baseline assessment. Accordingly, the effects of intensive care medicine and antibiotic therapy on EASIX were not considered. In addition, there was no superiority in terms of prognostic accuracy for EASIX in comparison with the established clinical scores SOFA and APACHE, with the highest combined sensitivity of 68.5% and specificity of 76.6% to predict 3-month mortality. Consequently, further prospective studies are needed to evaluate the diagnostic and prognostic potential of EASIX in patients with advanced liver disease in the intensive care setting more closely.

## 5. Conclusions

The present study is the first to assess the prognostic utility of EASIX in critically ill patients with LC. It indicated an association of ED with outcome, evidence of infection and renal function. EASIX has promising potential to exclude clinically apparent infections on ICU admission. EASIX is easy to calculate via routine laboratory parameters and does not contain subjective clinical interpretations. The score can be a valuable prognostic marker in a challenging population of critically ill patients with end-stage liver disease and ACLF and should be seen as complementary to established scores.

## Figures and Tables

**Table 1:** Baseline characteristics of the study population.

Baseline Characteristics of the Study Population	
**Female sex, N/total (%)**	69/188 (37%)
**Age, years**	59 (51–67)
**Body weight, kg**	75 (66–85)
**Body height, cm**	175 (168–180)
**Body mass index, kg/m^2^**	25 (22–27)
**SOFA**	11 (8–14)
**APACHE II**	22 (17–27)
**MELD**	26 (22–32)
**CTP**	12 (10–13)
**CTP C, N/total (%)**	154/188 (82%)
**No ACLF—Grade 0, N/total (%)**	23/188 (22%)
**ACLF, N/total (%)**	165/188 (88%)
**ACLF Grade I, N/total (%)**	39/165 (24%)
**ACLF Grade II, N/total (%)**	47/165 (28%)
**ACLF Grade III, N/total (%)**	79/165 (48%)
**CLIF-C OF, N = 188**	11 (9–14)
**CLIF-C ACLF, N = 165**	58 (50–65)
**CLIF-C AD, N = 23**	53 (47–59)
**Etiology of cirrhosis, N/total (%)**	Alcoholic 135/188 (72%)
	Viral 15/188 (8%)
	Autoimmune 9/188 (5%)
	Cryptogenic/NASH 29/188 (15%)
**Diagnoses at admission, N/total (%)**	Sepsis/Pneumonia 77/188 (41%)
	AKI/HRS 41/188 (22%)
	Gastrointestinal bleeding 38/188 (20%)
	Hepatic encephalopathy 32/188 (17%)
**Length of ICU stay, days**	11 (5–19)
**28-day mortality, N/total (%)**	85/188 (45%)
**3-month mortality, N/total (%)**	111/188 (59%)
**Clinical cause of death, N/total (%)**	Sepsis, Pneumonia 86/111 (77%)
	Gastrointestinal bleeding 13/111 (12%)
	Cardiocirculatory failure 12/111 (11%)
**Dialysis during ICU, N/total (%)**	105/188 (56%)
**LDH, IU/mL**	269 (209–366)
**Thrombocytes, 10^9^ cells/L**	73 (45–115)
**Creatinine, mg/dL**	1.9 (1.2–2.9)
**Bilirubin, mg/dL**	5.2 (2.2–12.6)
**INR**	1.8 (1.5–2.4)
**MAP, mmHg**	68 (62–75)
**Use of vasopressors, N/total (%)**	117/188 (62%)
**P_a_O_2_, mmHg**	86 (74–99)
**F_i_O_2_, %**	30 (30–40)
**Mechanical ventilation, N/total (%)**	84/188 (45%)
**HE, N/total (%)**	120/188 (64%)

**Table 2:** Correlations and linear regressions of baseline EASIX with conventional clinical scores and laboratory parameters on admission to the ICU.

	Spearman’s Coefficient r_s_	Linear Regression R^2^	*p*-Value
**APACHE-II**	0.592	0.262	**<0.001**
**SOFA**	0.625	0.262	**<0.001**
**MELD**	0.593	0.200	**<0.001**
**CTP**	0.438	0.093	**<0.001**
**ACLF Grade I–III**	0.477	0.109	**<0.001**
**CLIF-OF (N = 165)**	0.458	0.136	**<0.001**
**CLIF-ACLF (N = 165)**	0.380	0.083	**<0.001**
**CRP, mg/dL**	0.261	0.006	**<0.001**
**PCT, ng/mL**	0.456	0.054	**<0.001**
**WBC** **, 10^9^ cells/L**	0.084	0.022	0.250

## Data Availability

More detailed data are available upon request. To receive anonymised data, readers are welcome to contact the corresponding author.

## Supplementary Materials

The following supporting information can be downloaded at: https://www.mdpi.com/article/10.3390/jcm12072553/s1. Supplementary File S1: Percentage distribution of various types of infectious diseases with the corresponding EASIX levels on admission to intensive care unit.

## Abbreviations

**ACLF**	**Acute-on-chronic liver failure**
AlloTPL	Allogeneic stem cell transplantation
AUC	Area under curve
AKI	Acute kidney injury
APACHE-II	Acute and physiology chronic health evaluation II
BAL	Broncho-alveolar lavage
CRP	C-reactive protein
CTP	Child-Turcotte-Pugh
CT	Computed tomography
CLIF	Chronic liver failure
CLIF-OF	CLIF organ failure score
CLIF-ACLF	CLIF Acute-on-chronic liver failure score
COVID-19	Coronavirus disease 2019
ED	Endothelial dysfunction
EASL	European association for the study of the liver
EASIX	Endothelial activation and stress index
F_i_O_2_	Fraction of inspired oxygen
GvHD	Graft-versus-host disease
HE	Hepatic encephalopathy
HR	Hazard ratio
HRS	Hepatorenal syndrome
INR	International normalised ratio
IQR	Interquartile range
LC	Liver cirrhosis
MAP	Mean arterial pressure
**MELD**	Model of end-stage liver disease
**N**	**Number of patients**
**NASH**	**Non-alcoholic fatty liver disease**
NPV	Negative predictive value
**NO**	Nitric oxide
PPV	Positive predictive value
**PMN**	Polymorphonuclear neutrophils
P_a_O_2_	Arterial partial pressure of oxygen
PCT	Procalcitonin
R^2^	Linear regression coefficient
r_s_	Spearman’s coefficient
**ROC**	Receiver-operating-characteristic curves
SBP	Spontaneous bacterial peritonitis
SOFA	Sequential organ failure assessment
**SEC**	Sinusoidal endothelial cells
**WBC**	**White blood cell count (per microlitre)**
