# Supplementary material for: EASIX Is an Accurate and Easily Available Prognostic Score in Critically Ill Patients with Advanced Liver Disease
Source: J Clin Med. 2023 Mar 28;12(7):2553. doi: 10.3390/jcm12072553 (PMC10094870; doi:10.3390/jcm12072553)
Supplement: Supplementary file 1 [file jcm-12-02553-s001.zip › jcm-2264966-supplementary.pdf]

Supplementary File S1: Percentage distribution of different types of infectious diseases with corresponding EASIX-levels on admission to ICU.

		Percentage (fraction)	EASIX	p-value
	**"No infection"**	21% (39/188)	2.1 (1.3-4.3)	**< 0.001**
	**"Infection"**	79% (149/188)	9.7 (4.4-23.3)	
**Single infections**	**Respiratory**	26% (39/149)	11.5 (3.8-30.8)	
	**Urinary tract**	21% (31/149)	7.6 (4.1-18.3)	
	**SBP**	7% (10/149)	7.0 (3.5-11.5)	
	**Bacteremia**	5% (7/149)	9.8 (4.1-19.3)	
**Co-infections**	**Pneumonia + Urinary tract**	14% (21/149)	11.5 (5.6-30.5)	
	**Pneumonia + Bacteremia**	9% (13/149)	5.8 (3.5-19.8)	
	**SBP + Pneumonia + Urinary tract**	6% (9/149)	10.1 (4.3-26)	
	**SBP + Pneumonia**	4% (6/149)	8.8 (7.2-26.7)	
	**SBP + Bacteremia**	3% (5/149)	10.2 (8.6-25.6)	
	**Urinary tract + Bacteremia**	3% (5/149)	28.3 (12-39.7)	
	**SBP + Urinary tract**	2% (3/149)	47.7 (14.9-52.3)	
